# Comprehensive bioinformatics analyses of APOBECs family and identification of APOBEC3D as the unfavorable prognostic biomarker in clear cell renal cell carcinoma

**DOI:** 10.7150/jca.61972

**Published:** 2021-10-17

**Authors:** Zhenpeng Zhu, Xing Ji, Weijie Zhu, Tianyu Cai, Chunru Xu, Cong Huang, Shiming He, Yanqing Gong, Xuesong Li, Jian Lin, Liqun Zhou

**Affiliations:** 1 Department of Urology, Peking University First Hospital, Beijing 100034, China.; 2 Institution of Urology, Peking University, Beijing 100034, China.; 3 National Urological Cancer Center, Beijing 100034, China.; 4 Beijing Key Laboratory of Urogenital Diseases (Male) Molecular Diagnosis and Treatment Center, Beijing 100034, China.

**Keywords:** APOBEC family, clear cell Renal Cell Carcinoma, prognostic signature, overall survival, APOBEC3D

## Abstract

**Purpose:** At present, how early screening for ccRCC is still a thorny issue for urologists. Probing the mechanisms underlying the development of ccRCC and finding relevant prognostic biomarkers remains crucial. Therefore, we systematically analyzed the APOBEC family in this study and identified APOBEC3D as a prognostic biomarker.

**Methods:** In this study, based on the TCGA database, we systematically assessed the expression and prognosis of the APOBEC family and analyzed potential bioinformatic pathways. We then constructed nomograms to predict the prognosis of ccRCC patients better. Afterward, we further focused on APOBEC3D in our data on ccRCC specimens. The APOBEC3D should be extensively studied in ccRCC in the future.

**Results:** The results showed that the APOBEC family showed the most significant changes in expression in ccRCC. The pathway enrichment analysis showed that APOBEC3 family members mainly regulated cytidine and cytosine-related processes. Subsequently, the Cox regression was used to construct prognostic signature, and validated in ICGC and GEO databases. Next, a nomogram was created integrating clinical parameters showing good predictive performance. Finally, we screened for APOBEC3D and found in our clinical sample that patients with high expression of APOBEC3D had a worse prognosis.

**Conclusion:** Based on these results, APOBEC family members play important roles in the development of ccRCC, and APOBEC3D could serve as the biomarker for predicting patient prognosis.

## Introduction

Renal cell carcinoma (RCC) represents the 6^th^ most common malignancy in men and the 10^th^ in women, with high tumor heterogeneity and insensitivity to chemotherapy 1. Although with the improved medical technology, more RCC patients are detected at an early stage, distant metastases still occur in about 17% of those at first diagnosis 2. As the most common pathological type of RCC (70%-80%), clear cell renal cell carcinoma (ccRCC) is characterized by continuous genetic alteration such as VHL and PRBM1 3, 4. It is widely recognized that malignancies have a basis in the continuous genetic alteration that led to persistent activation of proliferative signaling and evasion of growth inhibition. Meanwhile, high-throughput sequencing has rapidly developed in the last two decades and can be widely applied for biomarker screening 5, 6. More importantly, two-thirds of genomic mutations occur randomly during the DNA replication process 5. Hence, exploring the expression and prognostic value of the gene family that affects DNA replication processes may provide new ideas and approaches to the treatment of ccRCC. Meanwhile, high-throughput sequencing has rapidly developed in the last two decades and can be widely applied for biomarker screening.

The APOBEC (apolipoprotein B mRNA editing catalytic polypeptide-like) family contains 11 members gradually identified as exogenous mutation factors 7, 8. Each family member has a specific role in DNA replication processes, involving binding nucleic acid and catalysis of cytidine to uridine deamination toward RNA or single-stranded DNA (ssDNA) 9. As critical regulators during transcription processes, the APOBEC genes could mediate the mutation of genomic ssDNA, affecting tumorigenesis and progression. Throughout human tumor genomes, APOBEC-mediated mutagenesis is pervasive 10. Increasing studies have shown the significant roles of APOBEC genes in many human tumors, notably in breast cancer, hepatocellular cancer, and ovarian cancer 11-13. In addition, the APOBEC family is strongly correlated with the tumor immune microenvironment and affects the tumor 14, 15.

Although the APOBEC family plays an essential role in tumorigenesis, the APOBEC family expression's comprehensive analysis and functions were still insufficient. In this study, using the transcriptome profile from the TCGA database, our clinical specimens, and online tools, we systematically explored the expression, prognostic value, and potential biological functions of the APOBEC family. We established the nomogram to predict the overall survival (OS) of ccRCC patients more accurately.

## Materials and Methods

### The transcriptome profile processing

The transcriptome profile (HTSeq-FPKM) and corresponding clinical data of ccRCC patients were obtained from The Cancer Genome Atlas (TCGA) (ccRCC; https://portal.gdc.cancer.gov/) cohort, Gene Expression Omnibus database (GEO) (GSE29609; http://www.ncbi.nlm.nih.gov/geo) and the International Cancer Genome Consortium (ICGC) (RCC; https://dcc.icgc.org/) public databases. We annotated the transcriptome profile using the Gencode (Version 26) GTF file and transformed the FPKM to TPM using RStudio (Version 3.6.3). Afterward, the RNA-seq profile in the TCGA database was used as the training cohort, and the RNA-seq profile in the ICGC database and GEO (GSE29609) database was used as the validation cohort. The clinical baseline of the patients in the TCGA database was shown in Supplementary Table 1.

### Specimens and cell lines

A total of 152 paired samples from patients diagnosed with clear cell renal cell carcinoma was used in this study, and the clinical baseline was shown in Supplementary Table 2. All patients underwent renal resection at Peking University First Hospital between June 2008 and January 2011. We obtained the clinical data of those patients from medical records. Moreover, this study was supported by the Ethics Committee of Peking University First Hospital, and written informed consent was obtained from these patients. All procedures were performed according to the World Medical Association Declaration of Helsinki.

The HK-2, 293, 786-O, 769-P, A498, ACHN, Caki-1, and OSRC2 were acquired from the American Type Culture Collection (ATCC, Manassas, VA, USA). Cells were cultured in DMEM (Invitrogen, Carlsbad, CA, USA) or 1640 (Invitrogen, Carlsbad, CA, USA) containing 10% fetal calf serum (Invitrogen) and 1% penicillin-streptomycin (Gibco). Cells were cultured in 10 mm culture dishes and 5% humidified atmosphere at 37 °C, and the medium was changed every 2-3 days.

### Identification of the prognostic differentially expressed APOBECs

Using the student t-test plugin in SPSS 26.0, we screened the differentially expressed APOBECs between ccRCC patients and normal controls. And then, univariate and multivariate Cox regression was used to screen prognostic APOBECs with ccRCC patients' OS. The P-value of genes less than 0.05 in multivariate Cox regression was identified as prognostic genes for further analysis.

### Biological functional enrichments and correlation analysis

The biological functional correlation of each APOBEC gene was estimated using the GeneMANIA database (http://www.genemania.org/) 16. Afterward, the ClueGO plugin from the Cytoscape software (Version 3.7.2) was used to explore the pathways network and determine their potential biological functions for the APOBECs genes, including Gene Ontology (GO) and Kyoto Encyclopedia of Genes and Genomes (KEGG) 17.

### Construction and validation of the APOBEC family-based signature

We established a prognostic signature with the significant APOBEC family genes based on the results during multivariate Cox regression. The prognostic signature formula is: Risk score = Exp (Gene1) × β (Gene1) + (Gene2) × β (Gene2) + … + (Genen) × β (Genen), where Exp represents the expression level and β represents the regression coefficient from the multivariate Cox regression signature. The patients from the database were divided into high- and low-risk groups according to the risk score's median levels with the calculated risk score, afterward, the prognostic performance of the risk score signature.

### Construction and validation of the predictive nomogram

To better predict the prognosis of patients with renal clear cell carcinoma, we established the predictive nomogram based on clinical parameters and prognostic signature. Briefly, we first performed univariate and multivariate Cox regression analyses to identify clinical parameters and riskscore that could be used as independent risk factors. Patients with a survival time of less than 30 days and the presence of unknown information (e.g., Nx, Gx) were excluded from this study. Subsequently, the significant factors were used to construct the predicted nomogram. We then evaluated the nomogram effect using calibration curves and time-dependent receiver operating characteristic (ROC) curves. The area under curve (AUC) value of 0.75 or higher was considered a significant predictive value, and the value of 0.60 or higher was regarded as acceptable for prediction.

### Immune infiltrate analysis

The relationship between immune cell infiltrate, and the expression of prognostic APOBECs was explored using the TIMER database (http://cistrome.shinyapps.io/timer/) 18. First, the abundances of six immunes infiltrate (B cells, CD4+ T cells, CD8+ T cells, Neutrophils, Macrophages, and Dendritic cells) are estimated by the TIMER algorithm. After, we used the TISIDB database to explore the association between the prognostic APOBECs expression and immune subtype in patients with ccRCC 19.

### Immunohistochemistry

Tissue sections were prepared from the paraffin-embedded tissue samples. Then immunostaining was performed using a two-step detection kit (Zsbio PV-9000, China). The sections were deparaffinized in xylene, rehydrated in a graded alcohol series, and then boiled in citrate buffer (pH 6.0) for 30 minutes in an autoclave. Endogenous peroxidase was blocked by incubation in 3% H2O2 and then washed in PBS, blocked with 10% goat serum (Zsbio, China) for 1 hour, and incubated with anti-APOBEC3D (K007649P, 1:2000, SolarBio). The sections were washed in PBS solution three times, incubated with a reaction enhancer kit for 20 minutes at room temperature, washed in PBS solution three times, and incubated with HRP-conjugated secondary antibody for 30 mins. All slides were counterstained with diaminobenzidine (DAB) solution and 20% hematoxylin and dehydrated. The primary antibody diluent was regarded as a negative control.

### Evaluation of immunostaining staining

Two experienced independent investigators examined all tumor slides by examining five random views and observing 100 cells per view at ×400 magnification. The staining intensity was classified as 0 (no staining), 1 (weak), 2 (moderate), 3 (strong). The proportion of stained tumour cells was scored as 0 (0%~5%), 1 (6%~25%), 2 (26%~50%), 3 (51%~75%), 4 (>75%). The multiplication of these two variables was calculated as final score: 0 (score 0-3); 1 (score 4-6); 2 (score 7-9); 3 (score 10-12) 20.

### Western blot analysis

Total proteins from cells were extracted in NP-40 lysis buffer and quantified by using the BCA method. Samples were transferred to polyvinylidene fluoride membranes and incubated overnight at 4 °C with antibodies against APOBEC3D (1: 1000; SolarBio) and β-actin (1:1000; Santa Cruz). After incubation with peroxidase-coupled anti-rabbit IgG (1:1000, Cell Signalling Technology) at 37 °C for 2 h, bound proteins were visualized using ECL (Pierce) and detected using a DNR Bioimaging System (DNR, Jerusalem, Israel). Relative protein levels were quantified using β-Actin as the loading control.

## Results

### Identification of the differentially expressed APOBECs

**Figure 1:**
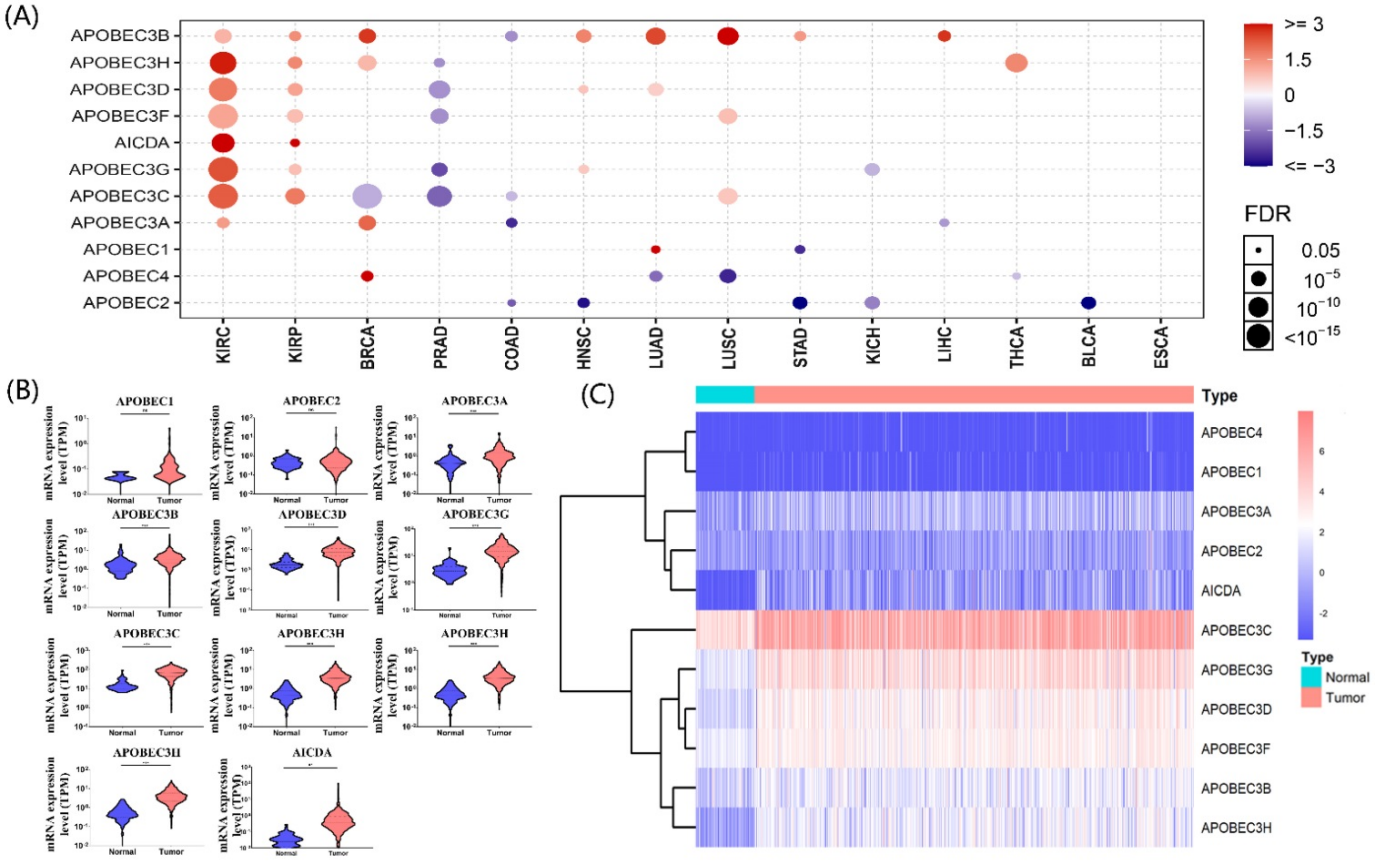
** Identification of the expression level of APOBEC family members. (A)** Results of a pan-cancer analysis of APOBEC family members. Red represents a positive correlation, while blue represents a negative correlation, the larger the point the more significant the p-value. Violin **(B)** and heatmap **(C)** of APOBEC family members between the ccRCC and normal controls in the TCGA cohort. “***”: p <0.001; “ns”: no significance.

The gene list of APOBECs was obtained from previous studies, and detailed information on APOBECs has been shown (Table 1). Using the Gene Set Cancer Analysis tool (GSCA; http://bioinfo.life.hust.edu.cn/web/GSCALite/), we explored the multi-omics APOBECs expression between tumor and paired normal samples among the TCGA database 21. Among them, the APOBECs expression showed the most significant alteration in ccRCC (Figure 1A). Hence, we further screened the differentially expressed APOBECs between ccRCC and normal controls in the TCGA-ccRCC cohort. As is shown in Figure 1B, the mRNA expression levels of more than half APOBECs were significantly elevated. To visualize the results clearly, we generated the heatmap using the R package (Figure 1C).

### Correlation and potential biological functions of APOBECs

**Figure 2:**
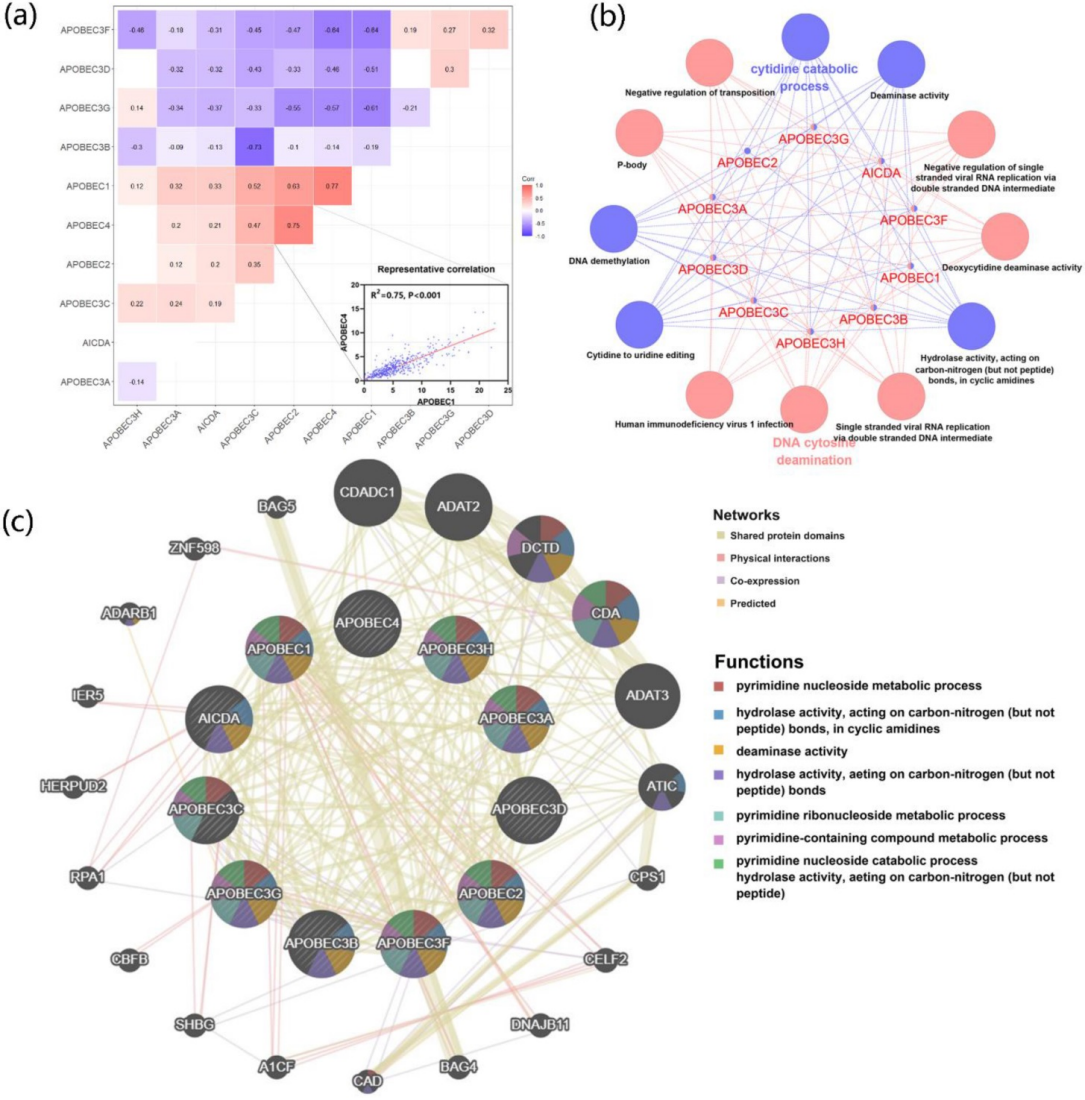
** The correlation of expression and biological functions of the APOBEC family members. (A)** Heatmap of correlations between the expression profiles of APOBEC family members, where the more significant ones have been shown. **(B)** GO and KEGG pathway enrichment of APOBEC3 family members visualized by the ClueGO plugin of Cytoscape. **(C)** Protein-protein interaction (PPI) network of APOBEC family members and their top 20 most significant correlated genes visualized by the GeneMANIA database.

To further explore the correlation and potential biological functions of the APOBECs family, first, we analyzed the Pearson correlation among each APOBECs mRNA expression level. The Pearson correlation coefficients of all APOBECs were calculated, and the most significant correlation was exhibited (Figure 2A). Afterward, the biological function enrichments were generated using the ClueGO plugin in Cytoscape. The GO and KEGG enrichment pathways with p <0.05 after the Benjamini-Hochberg procedure for false discovery rate (FDR) were considered significant (Figure 2B). The most significant biological enrichment pathways in two categories were Cytidine catabolic process and DNA cytosine deamination. Then, using the GeneMANIA tools, we explored the network and functions of APOBECs. Consistent with previous studies, the APOBECs family genes were significantly involved in mediating the pyrimidine processes and hydrolase activities (Figure 2C).

### Identification of the prognosis-related APOBECs in ccRCC patients

To screen the effects of APOBECs family genes expression on the ccRCC patients' overall survival, we implemented the univariate Cox regression and Kaplan-Meir analyses. APOBEC1 and APOBEC4 were excluded from the study because more than 50% of the TCGA database samples had 0 mRNA expression. In univariate Cox regression, high expression of APOBEC3G, APOBEC3D, APOBEC3B, and APOBEC3H was correlated with poor OS in ccRCC patients (Table 2). Similarly, in the Kaplan-Meir analyses, APOBEC3D and APOBEC3H were significantly related to the OS (Figure S1). Furthermore, multivariate Cox regression was condemned to screen the prognosis-related APOBECs in the TCGA cohort. The outcomes indicated that APOBEC3B (HR: 1.09), APOBEC3D (HR: 1.10), and APOBEC3F (HR: 0.84) exhibited independent prognostic value for ccRCC (Table 2).

### The correlation between the prognostic APOBECs and immune infiltration

**Figure 3:**
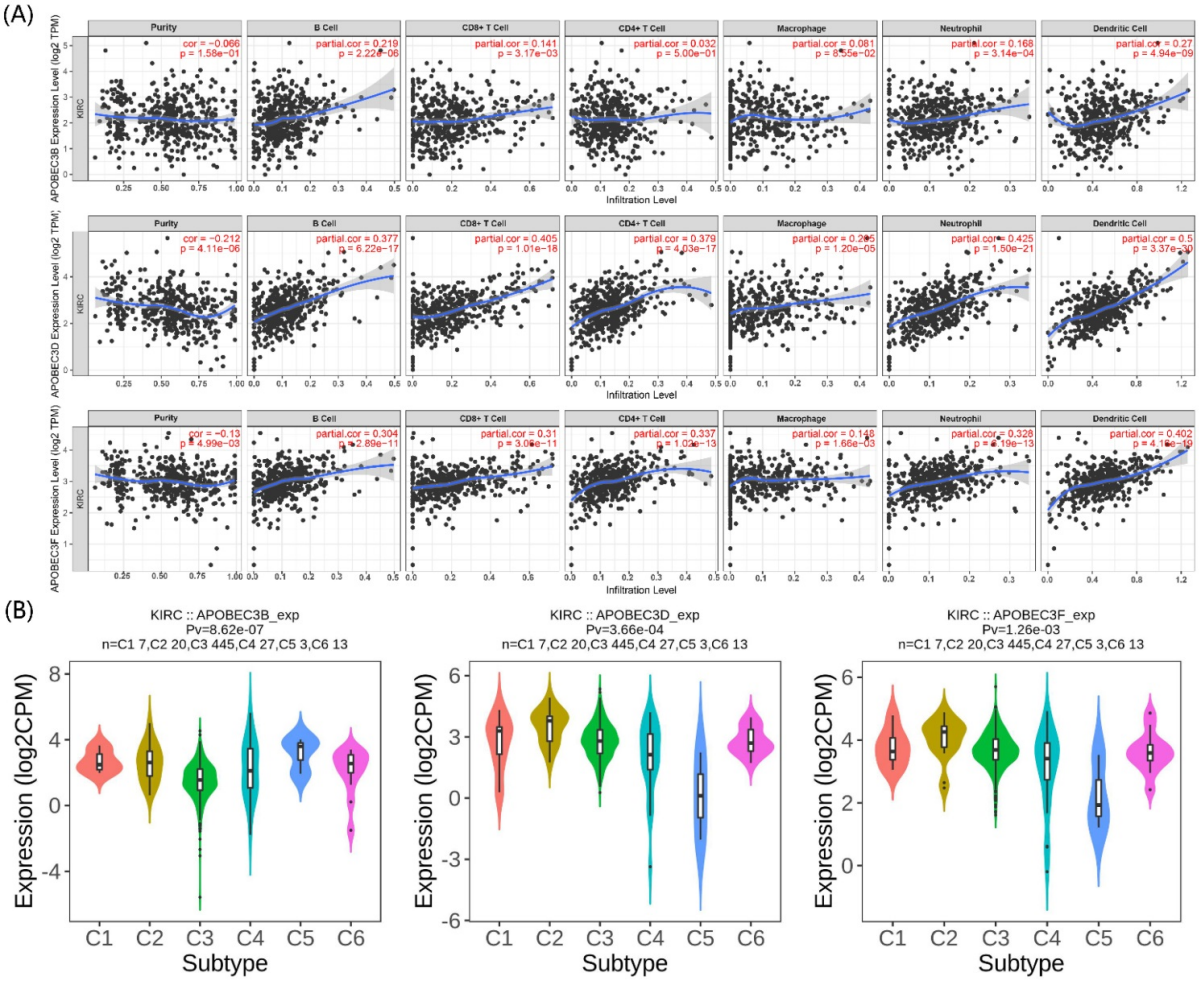
** Association between the prognostic APOBECs and immune infiltrates in the TCGA cohort. (A)** The correlation between the prognostic APOBECs and the immune cells validated by the TIMER database. **(B)** The correlation between prognostic APOBECs and immunophenotyping validated by the TISIDB database.

The immunity in the tumor microenvironment (TME) plays a vital role in developing the tumor. Hence, we explored the correlation between the prognostic APOBECs mRNA expression level and immune cells. Based on the TIMER database, the expression of all prognostic APOBECs was negatively correlated with tumor purity while positively correlated with B cells, CD8 T cells, CD4 T cells, Macrophages, Neutrophil, and Dendritic cells (Figure 3A). After that, we screened the correlation between the prognostic APOBECs expression level and the immune subtype of ccRCC. According to the outcome from the TISIDB database, the expression of all prognostic APOBECs was significantly altered between different immune subtypes of ccRCC (Figure 3B). The results above suggested that prognostic APOBECs were closely associated with immune infiltration in ccRCC.

### Construction and validation of the APOBECs based prognostic signature

**Figure 4:**
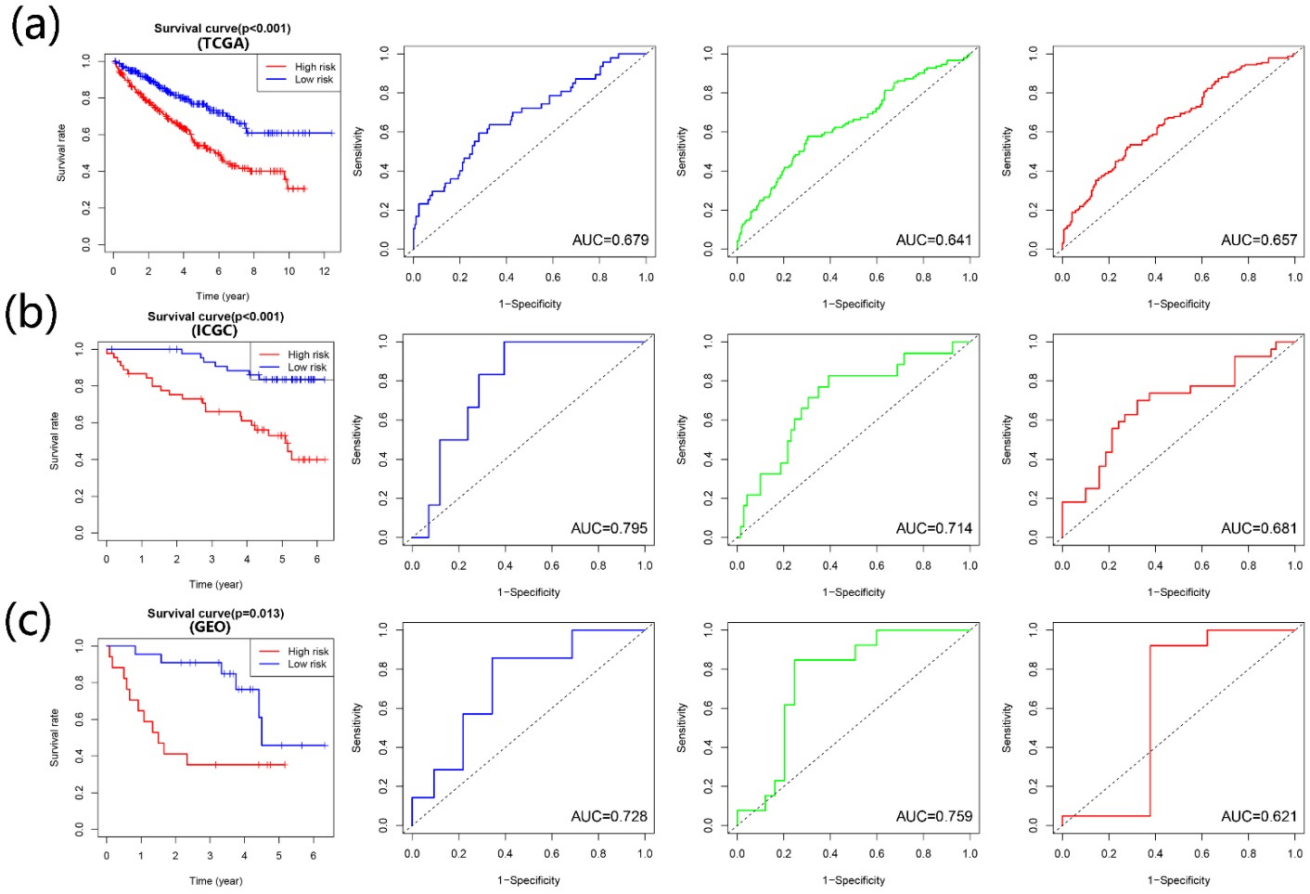
** Construction and validation of the risk signature based on the prognostic APOBECs.** The K-M survival plot and 1- (blue line), 3- (green line), and 5- (red line) year time-dependent ROC curves in the TCGA (**A**), ICGC (**B**), and GSE29609 (**C**) database.

According to the multivariate Cox regression outcome, four prognosis-related APOBECs were selected to establish the prognostic signature, including APOBEC3B, APOBEC3D, and APOBEC3F. The formula is risk score= APOBEC3B × 0.09915 + APOBEC3D× (-0.17722) + APOBEC3F × 0.08263. According to the median risk score, the patients with ccRCC were divided into the high- and low-risk groups. Kaplan-Meir analyses showed that high-risk patients tend to have a poorer prognosis than those with low-risk in the training cohort and validation cohort (Figure 4A-C). Furthermore, the time-dependent ROC curve showed that the prognostic signature had the favorable predictive ability of the 1-, 3-, and 5-year OS (Figure 4A-C).

### Construction and validation of the nomogram for prognostic evaluation

**Figure 5:**
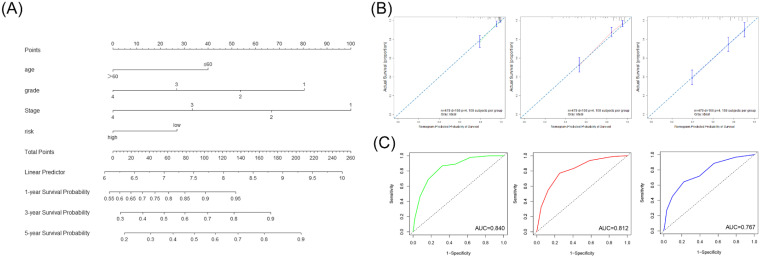
** Construction and validation of the nomogram based on the risk signature and clinical parameters. (A)** The prognostic nomogram was established to predict the 1-, 3-, and 5- year OS of the patients with ccRCC. **(B)** The calibration curve of the nomogram for validation at 1-, 3-, and 5- year OS. **(C)** The ROC curves for predicting the 1-, 3-, and 5- year OS.

We first screened whether prognostic signature could be used as an independent risk factor using the univariate and multivariate Cox regression analyses in the TCGA cohort (Table 3). The multivariate Cox regression analyses showed that the risk signature was significantly associated with the OS (HR=1.52). According to the outcome of multivariate Cox regression analyses, we combined the risk score and significant clinical factors to construct the nomogram model to predict the 1-, 3-, and 5-year OS in the TCGA cohort (Figure 5A). The C-index of the nomogram was 0.757. The calibration curve and ROC curve indicated that the nomogram could accurately predict the 1-, 3- and 5-year OS in patients with ccRCC (Figure 5B, 5C).

### The expression and prognostic value of APOBEC3D in specimens and cell lines

**Figure 6:**
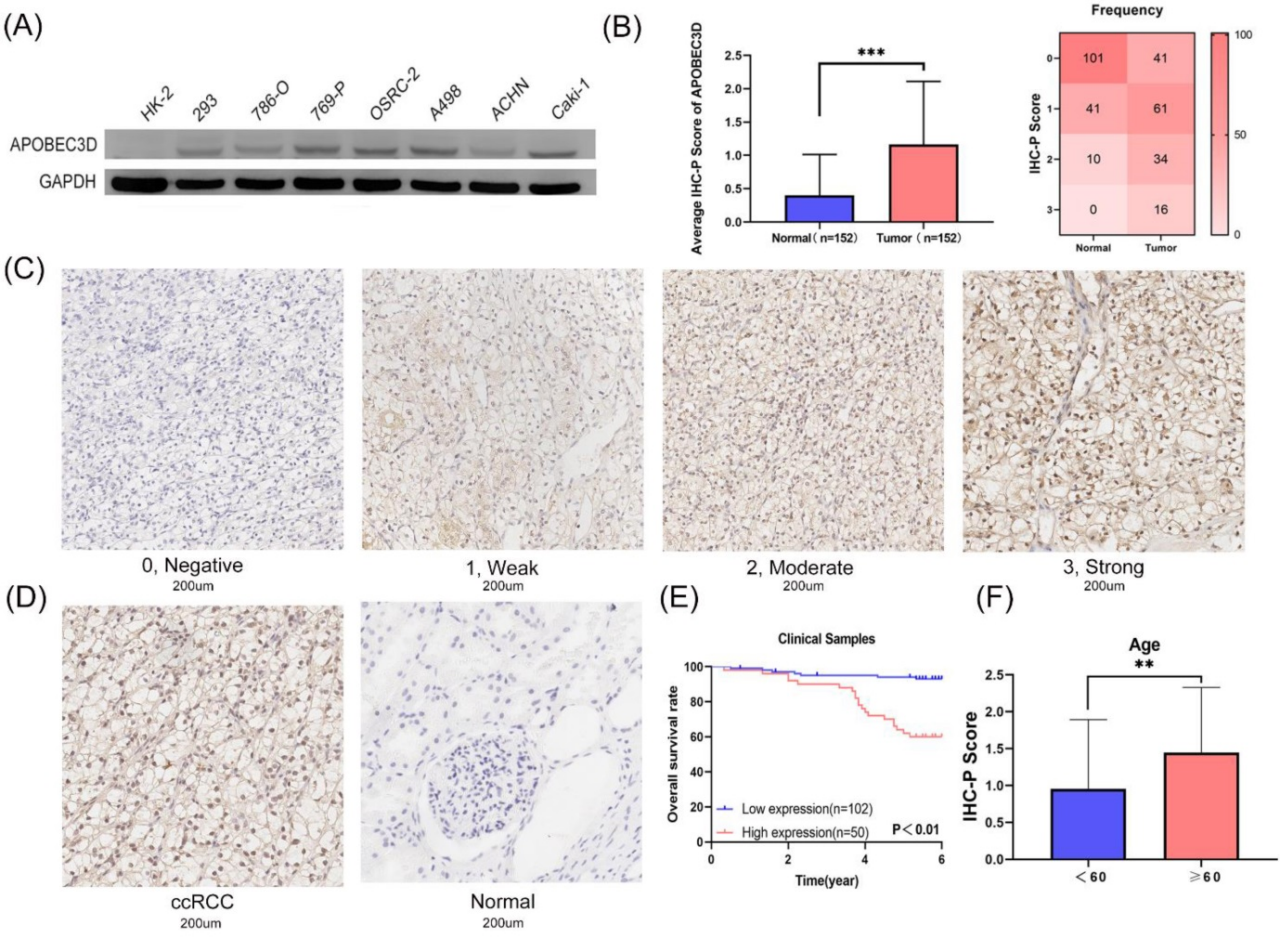
** Validation of the APOBEC3D in our cell lines and clinical specimens. (A)** The western blot analyses of the APOBEC3D expression in RCC and kidney cell lines. **(B)** The box plots and frequency heatmap of the IHC-P score between ccRCC and normal controls. **(C)** The four-degree representative images of the IHC-P score. **(E)** The K-M survival plot between the high and low APOBEC3D expression. **(E)** The correlation between the IHC-P score and age.

Based on these results above, we next explored the expression and prognostic value of APOBEC3D in clinical samples and cell lines. Only the APOBEC3D was significant in both Kaplan-Meir analyses and multivariate Cox regression analyses. Thus, we have further validated APOBEC3D in our clinical samples and cell lines. The APOBEC3D expression was elevated in RCC cell lines compared to normal kidney cell lines, especially in the A498 and Caki-1 cell lines (Figure 6A). We then performed IHC-P experiments in 152 paired samples. IHC-P Score was significantly higher in ccRCC than in normal controls. The frequency table for IHC-P scores is also shown (Figure 6B). The representative images of the IHC-P fraction were shown in Figure 6C and Figure 6D. K-M Plot showed that patients with high APOBEC3D expression had a poorer prognosis (Figure 6E). The APOBEC3D expression was closely related to patients' age (Figure 6F). Univariate and multivariate Cox regressions showed that APOBEC3D could be served as an independent factor in patients with ccRCC (Table 4).

## Discussion

As the third most common malignant tumor of the genitourinary system, ccRCC remains highly lethal with increasing incidence 22. Interestingly, epigenetic aberrations are commonly found in ccRCC, indicating the importance of epigenetic reprogramming in ccRCC 23. APOBEC family enzymes convert cytosine to uracil in ssDNA or RNA, which played critical roles in regulating epigenetic reprogramming processes 24-26. More importantly, the APOBEC family has emerged as a potential enzymatic source of mutation in cancers over the past decades. The APOBECs promote cancer development by affecting gene mutation and damage repair 27, 28. Previous studies have shown that APOBECs play essential functions in cancers, such as bladder cancer and breast cancer 29-31. However, few articles have explored the expression and prognostic value of the APOBECs in ccRCC. Hence, we comprehensively explored the expression and prognosis of the APOBECs based on the TCGA database.

We first explored the expression of the APOBECs in different cancer types using pan-cancer analysis. Expression alteration differences between tumor and normal controls were the most significant in ccRCC, especially APOBEC3 members. The results were consistent with a previous study, which indicated that APOBEC3 members caused mutations through DNA damage in kidney disease 32. We then explored the expression correlations and potential biological functions of APOBECs. The potential biological functions showed that APOBECs were correlated with the regulation of pyrimidine and the activity of related enzymes. These functions were in line with their reported functions 33. The KEGG enrichment in the ClueGO plugin of Cytoscape also confirmed the biological functions.

Subsequently, we explored the prognostic value of the APOBECs using the univariate and multivariate Cox regressions. Three prognostic APOBECs (APOBEC3B, APOBEC3D, and APOBEC3H) were identified. The immune microenvironment played important roles in the development of ccRCC, where the APOBECs showed significant regulatory functions 34, 35. We explored the correlation between the prognostic APOBECs and immune cells. The prognostic APOBECs were significantly associated with the CD8 T and CD4 T cells, which might affect the efficacy of immunotherapy for ccRCC. Previous studies reported the deletion of APOBEC3B influenced the immune activation in Asian women with breast cancer. Moreover, the APOBEC-enriched tumor was easily inclined to immune evasion 36. Thus, the APOBECs were closely related to the immune microenvironment, and the exact mechanisms need to be explored more in the future.

We then established and validated the prognostic signature based on the prognostic APOBECs using the Cox regression. The prognostic signature showed good predictive performance in both training and validation sets. Afterward, we conducted the nomogram integrating the prognostic signature and significant clinical parameters to better predict patients' performance with ccRCC. Again, nomograms show good predictive performance, especially for 1-year survival prediction.

Based on the above results, we further investigated APOBEC3D for its significance in multivariate Cox regression, K-M plot, and immune infiltration correlation. We then validated the expression of APOBEC3D in our clinical sample and on the prognosis. High expression of APOBEC3D meant poor prognosis. Due to the limited sample size, the correlation between APOBEC3D expression and grade and stage remains need to be explored. Univariate and multivariate Cox regressions showed that APOBEC3D could be served as an independent factor in patients with ccRCC. This gene was currently poorly studied and could be explored further in the future.

In the current study, there are still some flaws. First, the prediction model still needs to expand the sample size in future validation due to the online database's sample size limitation. Second, since the potential bioinformatic functions of prognostic APOBECs are all uncovered by the database, they need to be validated in future experiments.

Overall, we are the first to explore the expression and prognostic value of the APOBECs family in ccRCC and develop the prognostic, predictive signature. Based on these results, we focused on the significant APOBEC3D and validated it in our clinical samples and cell lines. Multicenter, larger-scale validation should be carried out in future clinical work.

## Supplementary Material

Supplementary figure and tables.

## Figures and Tables

**Table 1:** Detailed information of APOBEC family members

Gene symbol	Description	Location	Expression
AICDA	Activation Induced Cytidine Deaminase	12p13.31	n.s.
APOBEC1	Apolipoprotein B MRNA Editing Enzyme Catalytic Subunit 1	12p13.31	n.s.
APOBEC2	Apolipoprotein B MRNA Editing Enzyme Catalytic Subunit 2	6p21.1	n.s.
APOBEC3A	Apolipoprotein B MRNA Editing Enzyme Catalytic Subunit 3A	22q13.1	up-regulated
APOBEC3B	Apolipoprotein B MRNA Editing Enzyme Catalytic Subunit 3B	22q13.1	up-regulated
APOBEC3C	Apolipoprotein B MRNA Editing Enzyme Catalytic Subunit 3C	22q13.1	up-regulated
APOBEC3D	Apolipoprotein B MRNA Editing Enzyme Catalytic Subunit 3D	22q13.1	up-regulated
APOBEC3F	Apolipoprotein B MRNA Editing Enzyme Catalytic Subunit 3F	22q13.1	up-regulated
APOBEC3G	Apolipoprotein B MRNA Editing Enzyme Catalytic Subunit 3G	22q13.1	up-regulated
APOBEC3H	Apolipoprotein B MRNA Editing Enzyme Catalytic Subunit 3H	22q13.1	up-regulated
APOBEC4	Apolipoprotein B MRNA Editing Enzyme Catalytic Subunit 4	1q25.3	n.s.

**Table 2:** Univariate and multivariate Cox analyses of APOBEC family in the TCGA cohort

Gene	Univariate analysis		Multivariate analysis	
	HR (95%CI)	*p* value	HR (95%CI)	*p* value
APOBEC3G	1.014 (1.001, 1.026)	0.024	0.979 (0.950, 1.009)	0.163
APOBEC3D	1.037 (1.015, 1.059)	0.001	1.103 (1.047, 1.162)	**<0.001**
APOBEC3F	0.993 (0.950, 1.038)	0.760	0.857 (0.790, 0.929)	**<0.001**
APOBEC3A	1.070 (0.999, 1.147)	0.054	1.059 (0.989, 1.133)	0.099
APOBEC3B	1.070 (1.047, 1.094)	<0.001	1.084 (1.058, 1.112)	**<0.001**
APOBEC2	0.886 (0.696, 1.127)	0.325	0.889 (0.696, 1.134)	0.342
APOBEC3H	1.070 (1.031, 1.109)	<0.001	1.045 (0.982, 1.111)	0.165
APOBEC3C	1.003 (1.000, 1.007)	0.063	1.002 (0.996, 1.007)	0.588
AICDA	1.018 (0.999, 1.037)	0.057	1.010 (0.991, 1.029)	0.316

**Table 3:** Univariate and multivariate Cox analyses of clinical parameters and risk signature

Parameters	Univariate analysis		Multivariate analysis	
	HR (95%CI)	*p* value	HR (95%CI)	*p* value
gender	0.948 (0.690, 1.301)	0.740	0.840 (0.610, 1.161)	0.291
pM	4.360 (3.181, 5.978)	<0.001	1.506 (0.755, 3.001)	0.245
pT	1.871 (1.583, 2.210)	<0.001	0.878 (0.568, 1.360)	0.562
Stage	1.856 (1.622, 2.123)	<0.001	1.526 (0.954, 2.441)	0.078
Grade	2.227 (1.812, 2.738)	<0.001	1.485 (1.177, 1.874)	0.001
age	1.723 (1.260, 2.356)	<0.001	1.558 (1.135, 2.140)	0.006
risk	2.215 (1.596, 3.076)	<0.001	1.517 (1.077, 2.138)	0.017

**Table 4:** Univariate and multivariate Cox analyses of APOBECD in the our clinical cohort

Parameters	Univariate analysis		Multivariate analysis	
	HR(95%CI)	*p* value	HR(95%CI)	*p* value
Grade	2.427 (1.156, 5.093)	0.019	2.231 (0.930, 5.350)	0.072
Stage	1.382 (0.884, 2.160)	0.155	1.418 (0.835, 2.408)	0.196
Gender	1.085 (0.475, 2.478)	0.847	0.784 (0.330, 1.861)	0.581
Age	1.040 (1.006, 1.075)	0.021	1.036 (1.002, 1.069)	**0.033**
APOBEC3D	2.396 (1.616, 3.554)	<0.001	2.605 (1.697, 3.996)	**<0.001**

## Abbreviations

RCC: renal cell carcinoma
ccRCC: clear cell renal cell carcinoma
APOBEC: apolipoprotein B mRNA editing catalytic polypeptide-like
ssDNA: single-stranded DNA
OS: overall survival
TCGA: The Cancer Genome Atlas
GEO: Gene Expression Omnibus database
ICGC: International Cancer Genome Consortium
GO: Gene Ontology
KEGG: Kyoto Encyclopedia of Genes and Genomes
ROC: receiver operating characteristic
AUC: area under curve
DAB: diaminobenzidine
