# Supplementary material for: Comprehensive bioinformatics analyses of APOBECs family and identification of APOBEC3D as the unfavorable prognostic biomarker in clear cell renal cell carcinoma
Source: J Cancer. 2021 Oct 17;12(23):7101–10. doi: 10.7150/jca.61972 (PMC8558646; doi:10.7150/jca.61972)
Supplement: Supplementary file 1 — Supplementary figure and tables. [file jcav12p7101s1.pdf]

Supplementary files:

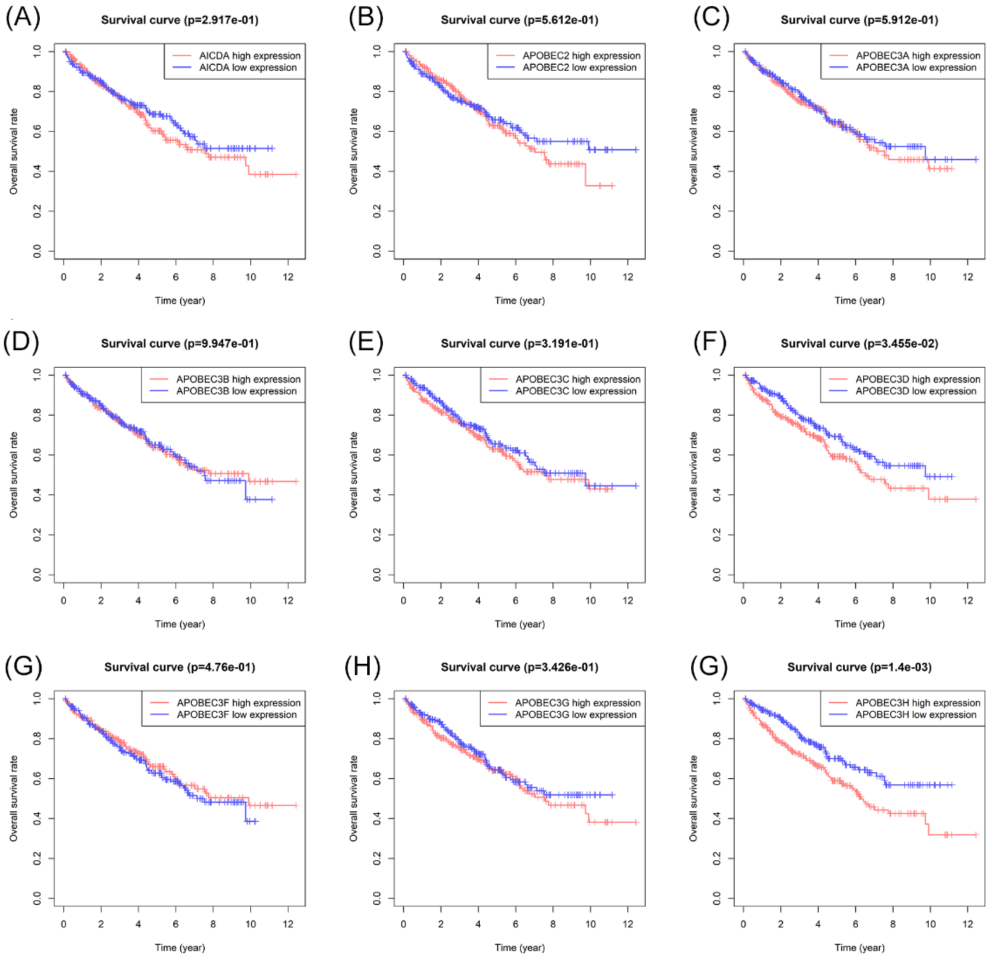

Supplementary Figure 1. K-M survival plot of the APOBEC family members. (A) AICDA; (B) APOBEC2; (C)APOBEC3A; (D)APOBEC3B; (E)APOBEC3C; (F)APOBEC3D; (G)APOBEC3F; (H)APOBEC3G; (I)APOBEC3H;

Supplementary Table 1 Clinical information of 530 ccRCC patients

Clinical parameters	Variable	Total (530)	Percentages (%)
Age	≤60	263	49.62
	>60	267	50.38
Gender	Male	344	64.91
	Female	186	35.09
Pathological T	T1	271	51.13
	T2	69	13.02
	T3	179	33.77
	T4	11	2.08
Pathological M	M0	420	79.25
	M1	78	14.72
	MX	32	6.04
Pathological N	N0	239	45.09
	N1	16	3.02
	NX	275	51.89
AJCC Stage	Stage I	265	50.00
	Stage II	57	10.75
	Stage III	123	23.21
	Stage IV	83	15.66
	Unkonwn	2	0.38
ISUP Grade	G1	14	2.64
	G2	227	42.83
	G3	206	38.87
	G4	75	14.15
	GX	8	1.51
Survival status	Dead	173	32.64
	Alive	357	67.36

Supplementary Table 2 Clinical information of 152 clinical samples

Clinical parameters	Variable	Total (152)	Percentages (%)
Age	≤60	91	59.87
	>60	61	40.13
Gender	Male	105	69.08
	Female	47	30.92
AJCC Stage	Stage I	121	79.61
	Stage II	7	4.60
	Stage III	24	15.79
	Stage IV	0	15.66
ISUP Grade	G1	35	23.02
	G2	105	69.08
	G3	12	7.90
Survival status	Dead	29	32.64
	Alive	123	67.36
